# Toxic Metals in Road Dust from Urban Industrial Complexes: Seasonal Distribution, Bioaccessibility and Integrated Health Risk Assessment Using Triangular Fuzzy Number

**DOI:** 10.3390/toxics13100842

**Published:** 2025-10-02

**Authors:** Yazhu Wang, Jinyuan Guo, Zhiguang Qu, Fei Li

**Affiliations:** 1 School of Business Administration, Zhongnan University of Economics and Law, Wuhan 430073, China; 202012100170@stu.zuel.edu.cn; 2 Research Center for Environment and Health, Zhongnan University of Economics and Law, Wuhan 430073, China; jinyuanguo@stu.zuel.edu.cn; 3 School of Information Engineering, Zhongnan University of Economics and Law, Wuhan 430073, China

**Keywords:** industrial complexes, land contamination, atmospheric sediment, toxic metal, exposure risk, bioaccessibility

## Abstract

Urban industrial complexes have been expanding worldwide, reducing the spatial separation between agricultural, residential, and industrial zones, particularly in developing nations. Urban road dust contamination, a sensitive indicator of urban environmental quality, primarily originates in urbanization and industrialization. Its detrimental impacts on human health arise not only from particulate matter itself but also from toxic and harmful substances embedded within dust particles. Toxic metals in road dust can pose health risks through inhalation, ingestion and contact. To investigate the seasonal patterns, bioaccessibility levels and the potential human health risks linked to toxic metals (Cadmium (Cd), Nickel (Ni), Arsenic (As), Lead (Pb), Zinc (Zn), Copper (Cu), and Chromium (Cr)), 34 dust samples were collected from key roads in proximity to representative industrial facilities in Wuhan’s Qingshan District. The study found that the concentrations of Cd, Pb, and Cu in road dust were within the limits set by the national standard (GB 15618-2018), while Ni and As were not. Seasonally, Ni, As, Pb, Zn, and Cr exhibited higher concentrations during the summer than in other seasons, whereas Cd levels were lowest in spring and highest in autumn, the opposite of Cu. According to the Simplified Bioaccessibility Extraction Test (SBET), the average bioaccessibility rates of toxic metals were Cd > Zn > Cu > Ni > Cr > As > Pb. An improved health risk assessment model was developed, integrating metal enrichment, bioaccessibility, and parameter uncertainty. Results indicated that Cd, Ni, Zn, Cu, As, and Cr posed no significant non-carcinogenic risk. However, for children, the carcinogenic risks of Cd and As were relatively high, identifying them as priority control metals. Therefore, it is recommended to periodically monitor As and Cd and regulate their potential emission sources, especially in winter and spring.

## 1. Introduction

With accelerated social and economic growth, urban areas have transformed into the most dynamic centers of human activity, and their ecological environment is influenced by industrial production, urban construction, transportation and residents’ living. Furthermore, urban industrial zones are being constructed on a global scale. This has led to a reduction in the significant geographical demarcation among agricultural, residential, industrial areas and the expansion of urban industrial complexes worldwide, particularly within developing nations. Urban road dust, also known as atmospheric sediment, serves as a significant sensitive indicator of local pollution levels [1]. Toxic metals (TMs), including cadmium (Cd), arsenic (As), lead (Pb), nickel (Ni), zinc (Zn), copper (Cu), chrome (Cr) and others, are characterized by unnatural degradation [2], which may damage the human respiratory [3], circulatory and digestive systems, and even cause cancer [4], mainly via inhalation of resuspended road dust. The toxic metals in road dust mainly originate from human activities, such as vehicle exhaust emissions, industrial production, construction work, agricultural operations involving chemical fertilizers, and daily traffic. Consequently, in areas with dense populations, toxic traffic flow, and concentrated industrial distribution, the content of toxic metals in road dust may be relatively high. In addition, meteorological factors like wind direction, temperature, and rainfall can affect the deposition, distribution, and concentration of toxic metals in road dust [5]. Therefore, it is crucial to assess the potential health risks posed by these metals through various exposure pathways.

The total concentration of toxic metals alone does not fully capture their potential adverse effects on ecosystems; this is better reflected by their bioaccessibility [6]. Relying solely on total TM concentrations for health risk assessments can lead to overestimation, as not all toxic metals are bioavailable for absorption [7]. Thus, incorporating bioaccessibility into traditional health risk models helps reduce uncertainty and provides a more accurate risk assessment. In addition, hotspots of road dust toxic metals pollution are often associated with urban core areas, major road intersections and areas near industrial zones. Wuhan, which functions as the core of Hubei Province, represents a crucial industrial center within China and is equipped with a complete industrial production framework. It is worthy of note that in Wuhan, the Qingshan District (QSD) is prominent for its iron and steel sectors. In 2023, the district’s GDP reached 100.33 billion RMB (approximately 14 billion USD as of 1 January 2024), with the secondary industry being dominant. Furthermore, QSD owns many small factories and related industrial parks, as well as urban areas, in or surrounding the main agricultural land [8], which is typical of an urban industrial complex. To some extent, local environmental pollution issues have been influenced by emissions from industrial activities and motor vehicle exhaust [9]. Hence, considering a comprehensive evaluation of metal accumulation, bioaccessibility, and the uncertainty of parameters, it is essential to construct an improved health risk assessment model for toxic metals in road dust. Subsequently, specific and targeted risk management strategies can be formulated and implemented in QSD.

The aims of this research were as follows: (1) to examine the seasonal variation in the occurrence of seven toxic metals (Cd, Ni, As, Pb, Zn, Cu, Cr) within the road dust of QSD throughout the years 2020 and 2021; (2) to develop and carry out an improved human health risk assessment integrating the Simplified Bioaccessibility Extraction Test (SBET) and triangular fuzzy number of toxic metals in road dust of a typical industrial area, QSD, in order to identify priority control toxic metals and areas; (3) to put forward targeted risk management suggestions combining seasonal and regional pollution risk characteristics.

## 2. Materials and Methods

### 2.1. Study Area

**Figure 1:**
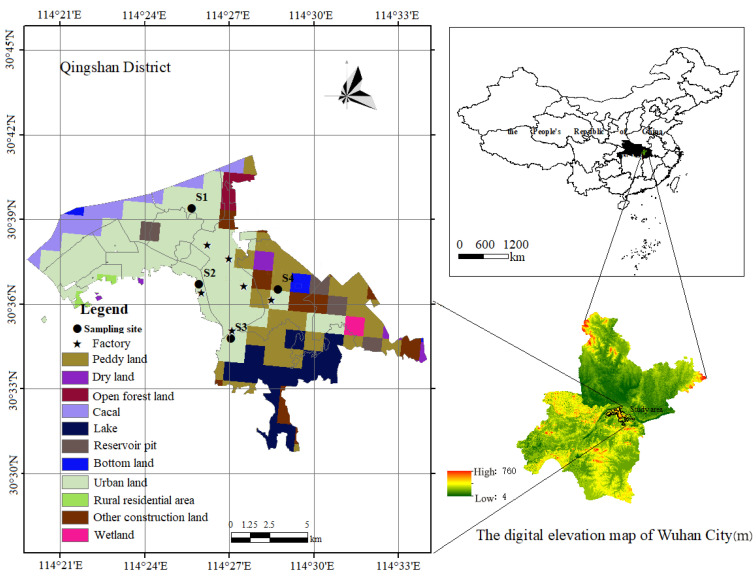
Sampling sites in Qingshan District. On the right are the map of China and the digital elevation map of Wuhan; on the left is the detailed map showing the sampling sites.

The Qingshan District is situated within the mid–low latitude zone. It falls under the climatic influence of the subtropical monsoon. This kind of climate endows the area with distinct characteristics. The district experiences relatively warm temperatures throughout the year, with marked seasonal variations in precipitation due to subtropical monsoons’ alternating wet and dry phases. Rainfall is primarily concentrated in the summer months, while the winter season is characterized by dry conditions and very little precipitation. This district has become a prominent industrial center, mainly because of its eight major industries, namely metallurgy, chemical manufacturing, environmental services, power generation, machinery, maritime transportation, construction, and building materials [10]. In 2019–2021, the secondary industry accounted for 67.5%, 64.6%, and 61.7% of the local GDP, respectively. Meanwhile, some large equipment enterprises (Figure 1) play a crucial role in the city’s manufacturing sector. These companies are complemented by a vibrant community of residential areas and educational institutions, which serve the employees and their families residing in the vicinity. Potential health hazards might be brought to the residents due to human exposure to toxic metals contained in the dust emitted during production and associated processes. To address this concern, four sampling sites were selected around these prominent industrial enterprises for detailed analysis.

### 2.2. Sample Collection and Analysis

Samples were collected at the four sites in QSD, including Shihua (S1), Changqian (S2), Wudong (S3) and Baiyushan (S4) (Figure 1), with consideration of the regional wind rose map. During the period spanning from the winter season of 2020 to the fall of 2021, samples were gathered at each site. To minimize the influence of meteorological factors on surface dust deposition, sampling was limited to periods following at least three consecutive days without rainfall and was carried out exclusively under clear, windless conditions. These criteria were implemented to reduce the effects of precipitation-induced wash-off and wind-driven resuspension, thereby enhancing the representativeness and comparability of samples across different seasons. Particulate samples were obtained from the region by means of a plastic brush and spade. Subsequently, these samples were meticulously blended to constitute a composite specimen, with the quantity collected from each site being guaranteed to reach a minimum of 0.5 kg. The samples which had been gathered were put into polyethylene bags for safe preservation and then were conveyed to the laboratory.

The total heavy metal content was determined using the HCl-HNO_3_-HF-HClO_4_ graphite furnace digestion method. Dust samples were accurately weighed into poly-tetrafluoroethylene digestion vessels and moistened with deionized water. The samples were then digested sequentially with 10 mL of HCl at 90 °C (until the volume was reduced to approximately 3 mL), followed by 5 mL of HNO_3_, 5 mL of HF, and 3 mL of HClO_4_ at 160 °C until the solution became clear. If digestion was incomplete, additional acids were added in the ratio of HNO_3_:HF:HClO_4_ = 3:3:1. After cooling, the digests were transferred to 50-mL volumetric flasks, with residues being dissolved in 1 mL of 1:1 HNO_3_ and 1 mL of 1:1 HCl. The solutions were then diluted to the final volume. The extracts were filtered through 0.45-μm membranes and stored at 4 °C for analysis. Quality control procedures included at least three blanks per batch and duplicate analyses for 10% of the samples. The total amount of each toxic metal element was measured by an Atomic Fluorescence Spectrometer (AFS-973) and Atomic Absorption Spectrophotometer (ZEEnit700P) in reference to HJ 787-2016 [11], HJ 752-2015 [12], HJ 540-2016 [13], HJ 786-2016 [14] and HJ 749-2015 [15]. Road dust primarily affects local populations through three exposure pathways: inhalation, ingestion, and skin contact. The bioavailability assessment of each exposure route requires customized evaluations considering material properties, environmental conditions, exposure duration, and human physiological characteristics. A study employing SBET method for arsenic (As) bioavailability in soil, supplemented by pig body experiments for validation, demonstrated exceptional accuracy in predicting toxic metal bioavailability. In this study, the SBET method was selected due to its extensive validation in environmental toxicology and health-related research, particularly for its ability to simulate gastric conditions relevant to human exposure. In vivo experiments are not only time-consuming and costly but also involve ethical concerns [16]. PBET and SBET are the two most used in vitro digestion methods for simulating human gastrointestinal digestion [17]. PBET is a two-step extraction method that replicates both the gastric and intestinal phases of human digestion, mimicking their respective environments and digestion times [18]. SBET is a simplified one-step extraction method, simulating human gastric conditions, the results of which were proved highly consistent with in vivo assays, such as swine models, thereby providing robust evidence of its predictive accuracy [19,20]. The reproducibility, methodological rigor, and feasibility of SBET to human health risk assessment make it particularly suitable for the bioaccessibility evaluation of toxic metals in road dust. The SBET method had been described in detail elsewhere [21]. SBET’s basic principle was to simulate the bioaccessible absorption dose of water-soluble toxic metals in the human stomach by controlling organic and inorganic components, pH, ambient temperatures of the extraction process and shock duration of simulated body fluids, so as to quantitatively identify the bioaccessibility of different toxic metals.

Throughout the sampling and testing process, we employed certified reagents for all chemical substances and ultrapure water for all solutions. Metal tools were not used; all glassware and plastic containers underwent immersion in 10% (*v*/*v*) nitric acid for at least 24 h, followed by multiple rinses with ultrapure water before use. To ensure the accuracy and reliability of analytical data, each sample batch included blank samples, duplicate samples, and National Reference Materials (GBW GSS-5). The deviation between duplicate sample groups remained below 5%, while reference material concentrations were within permissible ranges. Therefore, the testing data met the requirements of Chinese Soil Environment Monitoring Technical Specifications HJ/T 166-2004 [22].

### 2.3. Triangular Fuzzy Number

In environmental health risk assessment, inherent complexity and lack of clarity mean that simply employing mean concentration figures and single-parameter values in calculations may well give rise to inaccurate and one-sided conclusions [23]. Based on the literature review, triangular fuzzy number (TFN) and Monte Carlo simulation are widely recognized methods for uncertainty control. Compared with the Monte Carlo simulation method, the triangular fuzzy number has better applicability in processing imprecise and low-precision data [24]. Additionally, detailed investigation of exposure parameters can be time-consuming, labor-intensive, and challenging to implement due to budget constraints [25]. Consequently, this study employs TFNs for quantitative analysis and uncertainty control and discusses parameter sensitivity. A TFN is defined as a fuzzy number ${\tilde{A}}^{\alpha }$(*a*_1_, *a*_2_, *a*_3_) on the real number field R, and a membership function *μÃ*(x) is defined with the range of [0,1].

(1) $$\mu_{\tilde{A}}=\left\{\begin{array}{c} \begin{array}{c} 0\;\;\;\;\;\;\;\;\;\;\;\;\;\;\;x<a_{1}\\ \frac{x-a_{1}}{a_{2}-a_{1}}\;\;\;\;a_{1}\leq x<a_{2} \end{array}\\ \begin{array}{c} \frac{a_{3}-x}{a_{3}-a_{2}}\;\;\;\;a_{2}\leq x\leq a_{3}\\ 0\;\;\;\;\;\;\;\;\;\;\;\;\;\;\;\;x>a_{3} \end{array} \end{array}\right.$$

where *a*_1_, *a*_2_, and *a*_3_ are non-negative real numbers. Specifically, *a*_1_ represents the minimum value, *a*_2_ represents the maximum expected value, and *a*_3_ represents the maximum value. The α truncated set technique is used to streamline the computational process, and the calculation process is as follows:

(2) $${\tilde{A}}^{\alpha }=\left[a_{L}^{\alpha },a_{R}^{\alpha }\;\right]=\left[\left(a_{2}-a_{1}\right)\alpha +a_{1},\;-\left(a_{3}-a_{2}\right)\alpha +a_{3}\right]$$

where α is the confidence level, which is usually 0.9. ${\tilde{A}}^{\alpha }$ represents the number of fuzzy intervals of the fuzzy number $\tilde{A}$ under confidence. The four algorittoxic metals of fuzzy numbers are as follows [26]:

(3) $${\tilde{A}}_{1}^{\alpha }+{\tilde{A}}_{2}^{\alpha }=\left[a_{L1}^{\alpha }+a_{L2}^{\alpha },\;a_{R1}^{\alpha }+a_{R2}^{\alpha }\right]$$

(4) $${\tilde{A}}_{1}^{\alpha }÷{\tilde{A}}_{2}^{\alpha }=\left[a_{L1}^{\alpha }÷a_{R2}^{\alpha },\;a_{R1}^{\alpha }÷a_{L2}^{\alpha }\right]$$

(5) $${\tilde{A}}_{1}^{\alpha }\times {\tilde{A}}_{2}^{\alpha }=\left[a_{L1}^{\alpha }\times a_{L2}^{\alpha },\;a_{R1}^{\alpha }\times a_{R2}^{\alpha }\right]$$

(6) $${k\tilde{A}}_{1}^{\alpha }=\left[{ka}_{L1}^{\alpha },\;{ka}_{R1}^{\alpha }\right]$$

After the triangular fuzzy number is introduced, the resulting health risk is represented as a fuzzy interval, calculated using the formula provided above. The acceptable risk threshold for carcinogenic risk is often discussed compared to non-carcinogenic risk with a threshold (HQ<1). Generally, a carcinogenic risk (CR) below 1.0 × 10^−6^ is considered negligible, while a CR above 1.0 × 10^−4^ indicates a significant risk to the population [27,28]. To assist decision-makers in managing risk more intuitively, Li et al. (2017) [29] and Xu et al. (2018) [30] established a classification method for carcinogenic risk: I is a very low and negligible carcinogenic risk (−∞, 1.0 × 10^−6^); II is low carcinogenic risk, [1.0 × 10^−6^, 1.0 × 10^−5^); III is moderate carcinogenic risk, [1.0 × 10^−5^, 5.0 × 10^−5^); IV is high carcinogenic risk, [5.0 × 10^−5^, 1.0 × 10^−4^); V is very high carcinogenic risk, [1.0 × 10^−4^, 1). Relative to these risk levels [CRL*, CRR*], the membership function is defined as follows:

(7) $$M=\frac{\left|\left[{CR}_{L},{CR}_{R}\right]\cap \left[{CR}_{L}^{*},{CR}_{R}^{*}\right]\right|}{\left|\left[{CR}_{L},{CR}_{R}\right]\right|}\;$$

where *M* is the membership degree of the interval [*CR_L_*, *CR_R_*] to the risk level [CR_L_*, CR_R_*].

### 2.4. Fuzzy Health Risk Assessment Model

The main channels through which atmospheric sediments come into contact with the local population are ingestion via the mouth, inhalation through the respiratory system, and contact with the skin. Based on model settings of each exposure pathway by the Ministry of Ecology and Environment of China and USEPA, the classic exposure risk assessment model for toxic metals in road dust was established. Details of the classical health risk assessment model are provided in Supplementary Materials.

To further enhance scientific rigor and accuracy in assessing health risks associated with the oral ingestion pathway, the effects of metal enrichment and bioaccessibility were both under our consideration. $R_{D}$ is the bioaccessibility rate of toxic metals in road dust, which can be calculated by the ratio of the total contents of toxic metals ($C_{T}$) to the bioaccessible contents of toxic metals ($C_{DBA}$). In addition, under the consideration of parameter uncertainty influence, the sensitivity degrees of some human exposure parameters, such as exposure duration (ED), exposure frequency (EF), average time of exposure (AT), etc., which commonly refers to the standard default setting, are obviously low. In contrast, some other exposure parameters, such as pollution concentration (C), body weight (BW), and rate of dust intake into the body via the oral route (InhR), in addition to regional or seasonal variation, lead to much higher result sensitivity. This is consistent with some previous studies, which have demonstrated that exposure concentration typically accounts for most of the variance in risk (often exceeding 90%), while the contributions and duration are comparatively smaller [31,32]. Hence, the triangular fuzzy number of the sampled data was introduced and the seasonal toxic metals concentration in road dust was obtained, respectively. According to the investigation, the triangular fuzzy number was also calculated for other selected parameters (such as $\tilde{IngR}$ and $\tilde{BW}$), and the classic exposure risk assessment model was improved synthetically as follows:

(8) $${\tilde{INTAKE}}_{ing}=\frac{\tilde{C_{DBA}}\times ED\times EF\times \tilde{IngR}}{\tilde{BW}\times AT}\times 10^{-6}$$

(9) $${\tilde{INTAKE}}_{inh}=\frac{\tilde{C_{DBA}}\times ED\times EF\times \tilde{IinhR}}{\tilde{BW}\times AT\times PEF}$$

(10) $${\tilde{INTAKE}}_{dermal}=\frac{\tilde{C_{DBA}}\times ED\times EF\times SA\times SSAR\times ABS}{\tilde{BW}\times AT}\times 10^{-6}$$

(11) $$\tilde{HI}=\sum \tilde{{HQ}_{i}}=\sum \frac{\tilde{INTAKE}}{{RfD}_{i}}$$

(12) $$\tilde{CR}=\tilde{INTAKE}\times SF$$

where *HI* is the hazard index of bioaccessible toxic metals in road dust [33]. According to the USEPA, if the Hazard Index (HI) exceeds 1, there may be potential for non-carcinogenic risks. *HQ* is the hazard quotient of bioaccessible toxic metals in road dust. CR is the carcinogenic risk of bioaccessible toxic metals in road dust. *RfD* is the reference dose of toxic and harmful substances (mg/(kg·d)). *SF* is the carcinogenic slope factor (kg·d/mg). Parameters involved in the exposure model were selected mainly through HJ 25.3-2019 [34], HJ 25.1-2019 [35] and the domestic and foreign relevant studies [36,37,38,39], as shown in Supplementary Tables S1 and S2. Carcinogenic risk levels (Table S3) were classified according to the HJ 25.3-2019, USEPA and ICRP.

The membership function of the health risk level of toxic metals can be determined by interval statistical method combined with the classification standard of the health risk level. Assuming the interval of health risk levels is $\left[CR_{1},CR_{2}\right]$, then its degree of membership to the risk level $\left[CR_{1}^{*},CR_{2}^{*}\right]$ can be expressed as an equation.

(13) $$A(\lambda )=\frac{\left|\left[CR_{1},CR_{2}\right]\cap \left[CR_{1}^{*},CR_{2}^{*}\right]\right|}{\left[CR_{1}^{*},CR_{2}^{*}\right]}$$

where A(λ) is the membership of interval $\left[CR_{1},CR_{2}\right]$ to interval $\left[CR_{1}^{*},CR_{2}^{*}\right]$; $\left[CR_{1}^{*},CR_{2}^{*}\right]$ is the risk value range of the X-level risk level, X = I, II, III.

## 3. Results and Discussion

### 3.1. Characteristics of Toxic Metals in Road Dust

The overall concentrations of toxic metals measured in the road dust samples gathered from QSD were presented in Table S4. From the winter of 2020 to the autumn of 2021, the average contents of seven toxic metals in road dust were Cd 0.68 mg/kg, Ni 33.7 mg/kg, As 11.2 mg/kg, Pb 64.3 mg/kg, Zn 703 mg/kg, Cu 79.4 mg/kg and Cr 79.2 mg/kg. According to the toxic metal screening values outlined in the Soil Environmental Quality Risk Control Standard (GB 15618-2018) [40], the concentrations of Cd, Pb, and Cu were within the specified permissible limits. Statistical analysis confirmed the following findings: One-way ANOVA and Tukey HSD post hoc multiple comparisons of seven toxic metal concentrations revealed significant differences (F = 128, *p* < 0.001). Zinc (703 mg/kg) was 1033 times higher than cadmium (0.68 mg/kg), 62.9 times higher than arsenic (11.17 mg/kg), and 10.9 times higher than lead (64.30 mg/kg) (*p* < 0.001). Copper (79.37 mg/kg) and chromium (79.16 mg/kg) showed no significant difference (*p* = 0.982), but both were significantly higher than cadmium and arsenic (*p* < 0.01). On the basis of confirming the differences between TM types, we further analyzed their seasonal variability, and the results showed that most TMs exhibited significant seasonal concentration fluctuations (detailed as follows). The exceedance rate of Ni and As was 6% and 13%, and their maximum exceedance multiple was 1.01 and 1.17 times their standard values. For Ni and As with slight exceedances, the exceedance phenomenon was mainly observed in summer: the average concentration of Ni in summer reached 35.2 mg/kg (1.04 times the standard), and the average concentration of As in summer was 12.5 mg/kg (1.14 times the standard); in contrast, their concentrations in spring, autumn and winter were all within the standard limits, indicating a seasonal correlation of Ni and As exceedances. In the autumn and winter months, the atmospheric boundary layer is characteristically lower, which facilitates the formation of temperature inversions. Consequently, toxic metals (TMs) in particulate matter tend to accumulate in the atmosphere [41], leading to slower pollutant diffusion and potentially higher TM concentrations during these seasons [42]. Based on a variety of published studies from both domestic and international sources, it has been noted that the concentration of chemical elements in road dust particles tends to be elevated in urban industrial areas during the winter months [43,44]. The primary sources of toxic metals are identified as industrial activities and traffic emissions [45,46]. Cd has a connection with smelting activities or pollution caused by traffic. Therefore, the activities in the transportation sector make a significant contribution to the increased concentrations of Cu and Cd in the street dust of the QSD area [9]. The bioaccessibility rates of toxic metals in road dust were quantified (Table S5). The findings revealed the following average bioaccessibility rates: Cd (42.32%) > Zn (29.72%) > Cu (20.51%) > Ni (15.3%) > Cr (6.87%) > As (6.2%) > Pb (3.1%).

### 3.2. Health Risks of Toxic Metals in Road Dust

#### 3.2.1. Non-Carcinogenic Risk of Toxic Metals in Road Dust

**Figure 2:**
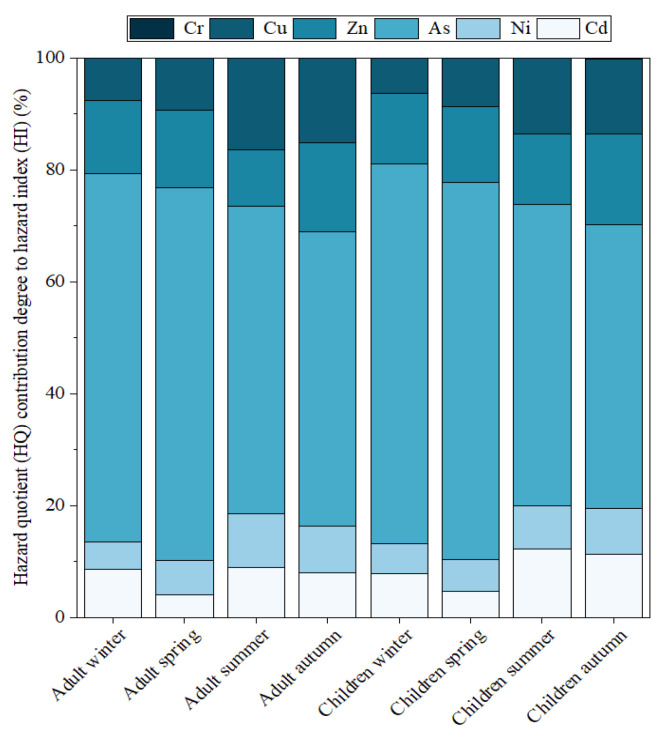
Different populations’ (adults, children) and seasons’ HQ contribution degree to HI of toxic metals (Cr, Cu, Zn, As, Ni, Cd).

The hazard quotients (HQs) regarding adults and children from winter 2020 to autumn 2021 were calculated and are presented in Table S6. For both adult and pediatric populations, the HQs for Cd, Ni, As, Zn, Cu, and Cr all remained below 1. This indicates that there is not a substantial risk of non-carcinogenic impacts caused by these metals. The hazard indexes (HIs) for both adults and children remained below 1 across all seasons, with the highest relative risk of non-carcinogenic effects observed in spring, as detailed in Table S7. The HQ contribution of each TM was shown in Figure 2. Notably, As consistently had the largest contribution in all seasons. Geographically, the analysis indicated that the HQ values for As were particularly elevated at sampling point S2, highlighting this area as a potential hotspot due to nearby sources of TM emissions.

#### 3.2.2. Carcinogenic Risk of Toxic Metals in Road Dust

**Figure 3:**
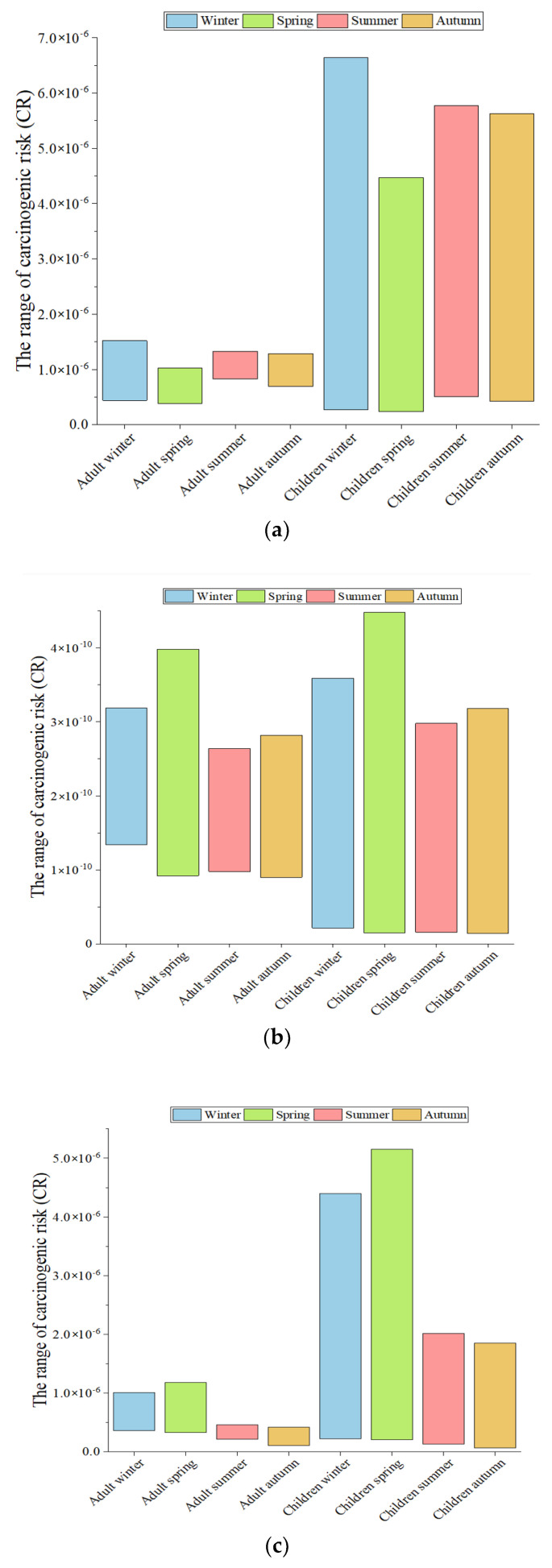

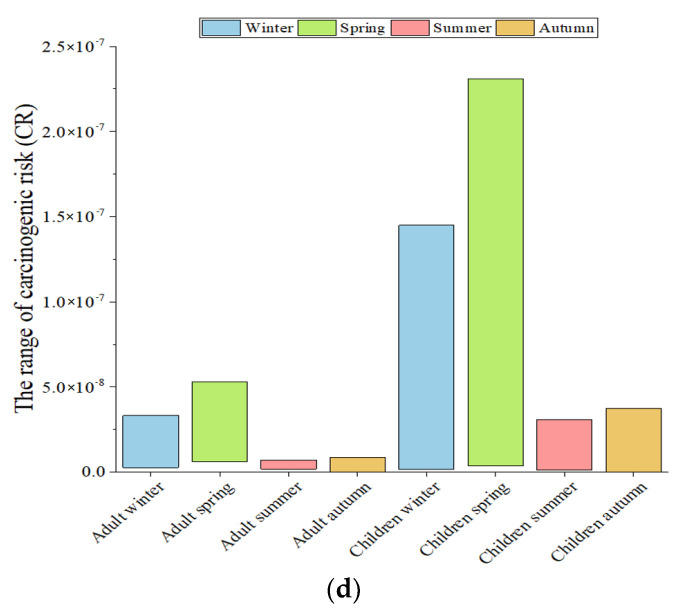
Under different populations (adults, children) and seasons carcinogenic risk of toxic metals via their main exposure pathway. (**a**) Carcinogenic risk of Cd via oral ingestion pathway. (**b**) Carcinogenic risk of Ni via respiratory inhalation pathway. (**c**) Carcinogenic risk of As via oral ingestion pathway. (**d**) Carcinogenic risk of Pb via oral ingestion pathway.

Based on Equations (4)–(6) and (8), the carcinogenic risks (CRs) associated with Cd, Ni, As, and Pb through three exposure pathways were calculated, as shown in Table S8. According to the maximum membership principle, the total CR for Ni, As, and Pb in adults via the three pathways was classified as level I, indicating no risk. Conversely, the total CR for Cd and As in children was categorized as level II, signifying a medium risk. Among the various exposure pathways, it was determined that ingestion through the mouth was the principal way for Cd, As, and Pb to enter the body. Notably, the CRs for Cd and As at the S2 sampling site were generally higher, with their maximal CRs exceeding 1 × 10^−6^ through this pathway, as illustrated in Figure 3. Generally, Cd and As are associated with smelting operations or transportation-related pollution [10]. Furthermore, their presence may be attributed to the deposition and accumulation of industrial dust around WISCO’s auxiliary facilities, such as the coking plants, fly ash plants, and machinery manufacturing plants [47].

#### 3.2.3. Comprehensive Analysis and Suggestions

SBET was used to identify priority control toxic metals, though the results varied across different components and extraction procedures [48,49]. Consequently, an improved health risk assessment model for toxic metals in road dust was developed, which incorporated factors such as metal enrichment, bioaccessibility, and parameter uncertainty. The analysis identified As and Cd as the key toxic metals requiring priority control measures in QSD. Marija [50] et al. evaluated the inhalation bioaccessibility of potentially toxic elements (PTE) in high-concentration dust from mining–smelting and traffic-related pollution sources. They found that the average inhalation bioaccessibility of zinc (Zn), lead (Pb), antimony (Sb), cadmium (Cd), and manganese (Mn) in artificial lung fluid (ALF) solution ranged from 40% to 51.5%. Han’s [51] team analyzed ten toxic metals in road dust from Anyang and observed significant accumulation of manganese (Mn), zinc (Zn), lead (Pb), cadmium (Cd), and copper (Cu), with Cd identified as the priority pollutant. Additionally, except for Zn in the gastric fluid phase, the gastrointestinal bioaccessibility of other toxic metals was mostly below 20%. The conclusions of this study—that Cd is a priority control metal and Zn has high bioaccessibility—are consistent with the aforementioned research. Among the diverse exposure channels, the intake via the oral route was identified as the foremost factor in elevating the levels of carcinogenic risk (CR). This underlines its significance and calls for enhanced focus and control measures. Furthermore, As and Cd in road dust from QSD are primarily derived from combined pollution sources—traffic and industry [10]. Considering the land use characteristics surrounding the area, the sampling point S2 was notably close to the Western Iron and Steel Center (WISC). Typically, steel production in our country involves processing iron ore through several redox reactions, generating smoke laden with toxic metals [52]. This smoke, carried by prevailing winds, is likely responsible for the elevated HQ and CR levels observed at the S2 site. Additionally, the wind rose diagram (Figure 1) illustrates that QSD is predominantly affected by the northeast wind, with residential areas situated southwest of S2. Given these conditions, it is essential to prioritize the management and reduction of TM emissions from industrial sources, within residential areas and along the two sides of surrounding roads, plant greening plants and toxic metal-accumulating plants. Additionally, it is important to specify the road cleaning frequency for different functional areas (e.g., roads around industrial zones should undergo mechanical cleaning twice a day and sprinkler irrigation three times a day, while roads in residential areas should be cleaned once a day and sprinkled with water twice a day). In addition, require cleaning vehicles to be equipped with integrated “dust suction + water sprinkling” equipment to avoid secondary diffusion of fugitive dust. Local residents should particularly pay attention to As and Cd pollution in dust, especially since the industrial zone consistently exhibits higher TM concentrations compared to other areas. Attention should be paid to the protective measures for vulnerable groups, such as children and the elderly (e.g., children should wash their hands promptly after outdoor play and avoid eating with their hands; the elderly should wear dust-proof masks during morning exercise; families should wipe the ground and furniture with a wet cloth regularly to reduce dust accumulation), and popularize them through community bulletin boards, public accounts, door-to-door publicity and other forms. Additionally, with regard to the feasibility of the proposed risk management measures, such as enhancing road cleaning and greening, it is important to make those actions align with the local urban development plan. For instance, the Wuhan Qingshan District’s “14th Five-Year Plan” for road network and landscaping construction explicitly sets quantitative targets for greening rates and park areas, while also allocating government funds and establishing financing mechanisms to ensure successful implementation. It could make sure those suggestions are feasible, contributing to local sustainable development benefits.

## 4. Conclusions

This study examined the seasonal fluctuations, bioavailability, and potential human health risks associated with toxic metals in the road dust of Wuhan’s QSD. According to GB 15618-2018, the contents of Cd, Pb and Cu were within acceptable limits. However, Ni and As exceeded thresholds in 6% and 13% of all the samples, respectively. The seasonal distribution demonstrated that the levels of Ni, As, Pb, Zn and Cr were relatively greater during the summer as opposed to other seasons. In contrast, Cd levels were lowest in the spring and peaked in the autumn, which was the reverse pattern observed for Cu. The average bioaccessibility rates followed the sequence: Cd > Zn > Cu > Ni > Cr > As > Pb, showing no significant seasonal or regional variations. Furthermore, the non-carcinogenic risk levels for Cd, Ni, Zn, Cu, As, and Cr were not significant. However, carcinogenic risks for Cd and As in children were considered medium, while Ni, As, and Pb posed no carcinogenic risk to adults. Consequently, Cd and As were determined to be priority metals for control measures. Risk management strategies specifically tailored to the findings from the distribution analysis and health risk assessments include the following: (1) Implementing individual protective measures against key exposure pathways, prioritizing the reduction of atmospheric particulate matter inhalation over hand–oral dust intake. Efforts should focus on enhancing controls at the S2 sampling site and nearby high-polluting enterprises and optimizing steel production processes and waste gas treatments to minimize toxic metal emissions. (2) Enhancing monitoring and controlling the sources of metals, particularly As and Cd. This involves continuous monitoring and controlling pollutant emissions during industrial production, especially during the winter and spring. Long-term and regular tracking of indices is also recommended to boost the effectiveness of risk monitoring in QSD. (3) Based on the findings from local receptor behavior surveys, environmental risk education should inform residents near the identified enterprises about personal protective measures to enhance their health and safety. Information on additional forms of pollution should be disseminated through various channels to ensure it reaches as many people as possible. (4) Establish a trinity monitoring system covering “road dust–soil–vegetation”. Collect dust, soil and plant samples monthly from Site S2 and surrounding key locations (such as industrial zone exits, residential area entrances and school playgrounds), and test the concentrations and bioaccessibility of arsenic (As) and cadmium (Cd). Evaluate the effectiveness of remediation measures quarterly and dynamically adjust the remediation plan based on the monitoring results.

## Data Availability

The original contributions presented in this study are included in the article and Supplementary Materials. Further inquiries can be directed to the corresponding authors.

## Supplementary Materials

The following supporting information can be downloaded at: https://www.mdpi.com/article/10.3390/toxics13100842/s1, Table S1: Selection of exposure parameters; Table S2: Value of reference dose for non-carcinogenic heavy metals (RfD) and carcinogenic slope factor (SF) via different exposure pathways; Table S3: Carcinogenic risk (CR) assessment criteria; Table S4: Total contents of heavy metals in road dust; Table S5: The bioaccessibility rates of heavy metals (RD) in road dust; Table S6: Hazard quotient (HQ) of heavy metals in road dust; Table S7: Hazard index (HI) of heavy metals in road dust; Table S8: Carcinogenic risk of heavy metals in road dust via different pathways.
