# Supplementary material for: Toxic Metals in Road Dust from Urban Industrial Complexes: Seasonal Distribution, Bioaccessibility and Integrated Health Risk Assessment Using Triangular Fuzzy Number
Source: Toxics. 2025 Oct 2;13(10):842. doi: 10.3390/toxics13100842 (PMC12567818; doi:10.3390/toxics13100842)
Supplement: Supplementary file 1 [file toxics-13-00842-s001.zip › toxics-3872501-supplementary.pdf]

Supplementary Materials

Table S1. Selection of exposure parameters

Parameters	Units	Adults	Children
ED	a	24	6
EF	d/a	350	350
IngR	mg/d	100	200
BW	kg	61.8	19.2
AT (carcinogenic)	d	27740	27740
AT (non-carcinogenic)	d	2190	2190
IinhR	m^3/d	14.5	7.5
PEF	m^3/kg	1.32×10^9	1.32×10^9
SA	cm^2/d	4925	2556
SSAR	mg/cm^2	0.07	0.2
ABS	dimensionless	0.001(As is 0.03)	0.001(As is 0.03)

Table S2. Value of reference dose for non-carcinogenic heavy metals (*RfD*) and carcinogenic slope factor (*SF*) via different exposure pathways

Heavy metals	RfD_o	RfD_i	RfD_d	SF_o	SF_i	SF_d
Cd	0.001	0.0000026	0.000025	6.1	0.0018	6.1
Ni	0.02	0.000023	0.0008	-	0.84	-
As	0.0003	0.0000038	0.0003	1.5	3.3	3.66
Pb	-	-	-	0.0085	0.042	-
Zn	0.3	-	0.3	-	-	-
Cu	0.04	-	0.04	-	-	-
Cr-III	1.5	-	0.0195	-	-	-

RfD_o, RfD_i, RfD_d are the oral ingestion, respiratory inhalation, dermal contact reference dose respectively, $mg\cdot(kg\cdot d)^{-1}$. SF_o, SF_i, SF_d are the oral ingestion, respiratory inhalation, dermal contact carcinogenic slope factor respectively, $kg\cdot d\cdot mg^{-1}$. "–" indicates no related data.

Table S3. Carcinogenic risk (CR) assessment criteria

Level	Risk	Value-at-risk (CR)
Class I risk	No risk	< 1.00E-06
Class II risk	Medium risk	[1.00E-06, 1.00E-05]
Class III risk	High risk	>1.00E-05

Table S4. Total contents of heavy metals in road dust mg/kg

Seasons	Sites	Cd	Ni	As	Pb	Zn	Cu	Cr
Winter	S1	0.54	20.28	12.24	40.41	203	26.93	44.27
	S2	0.91	32.04	19	85.09	440	334.97	57.15
	S3	0.64	42.61	19.15	62.89	349	63.11	68.47
	S4	0.64	21.26	21.18	54.09	317	38.85	73.82
Spring	S1	0.45	17.59	12.75	49.58	315	31.73	49.81
	S2	1.06	151.81	23.45	126.92	1190	167.28	285.69
	S3	0.66	39.92	18.81	64.77	319	102.83	88.26
	S4	0.55	26.77	12.78	52	178	36.24	76.35
Summer	S1	0.81	18.74	3.96	34.12	107	23.16	96.28
	S2	0.66	28.93	5.42	48.08	284	147.99	45.41
	S3	0.64	13.5	4.09	38.45	1600	48.69	24.85
	S4	0.54	16.65	4.2	31.4	51.4	46.98	76.57
Autumn	S1	0.67	32.79	5.1	74.69	796	28.78	75.86
	S2	0.57	36.09	5.64	142.39	4090	42.83	95.23
	S3	0.72	20.77	5.72	86.32	514	44.57	52.64
	S4	0.76	18.82	5.31	37.6	500	84.99	55.87

Table S5. The bioaccessibility rates of heavy metals (R_D) in road dust

Seasons	Sites	Cd	Ni	As	Pb	Zn	Cu	Cr
Winter	S1	29.63%	15.78%	5.96%	2.28%	44.29%	12.11%	10.05%
	S2	50.55%	19.85%	6.53%	8.46%	55.00%	3.62%	7.45%
	S3	34.38%	13.56%	3.19%	2.13%	30.09%	17.13%	6.44%
	S4	32.81%	18.72%	2.55%	1.26%	26.09%	41.39%	5.69%
Spring	S1	37.78%	12.51%	5.10%	4.86%	20.73%	12.92%	8.17%
	S2	29.25%	5.22%	6.18%	9.04%	24.29%	14.69%	1.73%
	S3	39.39%	16.58%	3.83%	3.24%	53.61%	13.16%	5.39%
	S4	25.45%	18.49%	3.83%	2.98%	43.09%	28.53%	5.41%
Summer	S1	43.21%	12.49%	8.08%	2.17%	-	38.73%	3.96%
	S2	59.09%	18.22%	10.52%	3.20%	31.97%	4.62%	8.39%
	S3	62.50%	24.89%	13.20%	3.93%	7.88%	17.58%	17.42%
	S4	55.56%	15.44%	8.57%	1.50%	-	39.70%	4.44%

Autumn	S1	44.78%	6.56%	6.47%	1.50%	8.00%	31.41%	4.72%
	S2	43.86%	7.29%	9.22%	1.30%	2.98%	10.27%	5.20%
	S3	37.50%	27.11%	2.80%	0.05%	32.10%	19.14%	9.92%
	S4	51.32%	12.06%	3.20%	1.78%	36.00%	23.16%	5.53%

"–" indicates the result is invalid.

Table S6. Hazard quotient (HQ) of heavy metals in road dust

Heavy metals	Seasons	Adults	Children
Cd	Winter	[0.001084 , 0.003736]	[0.000633 , 0.015344]
	Spring	[0.000948 , 0.002517]	[0.000553 , 0.010341]
	Summer	[0.002032 , 0.003248]	[0.001186 , 0.013343]
	Autumn	[0.001693 , 0.003167]	[0.000988 , 0.013009]
Ni	Winter	[0.001085 , 0.002586]	[0.000619 , 0.010373]
	Spring	[0.000746 , 0.003185]	[0.000425 , 0.012265]
	Summer	[0.000794 , 0.002117]	[0.000452 , 0.008151]
	Autumn	[0.000729 , 0.002261]	[0.000416 , 0.009183]
As	Winter	[0.011489 , 0.031631]	[0.006897 , 0.133634]
	Spring	[0.010425 , 0.036416]	[0.006259 , 0.146476]
	Summer	[0.006808 , 0.014315]	[0.004087 , 0.05758]
	Autumn	[0.003404 , 0.01306]	[0.002044 , 0.05604]
Zn	Winter	[0.00158 , 0.00557]	[0.000982 , 0.024234]
	Spring	[0.00125 , 0.006648]	[0.000775 , 0.028876]
	Summer	[0.00174 , 0.003128]	[0.001078 , 0.013589]
	Autumn	[0.00122 , 0.004141]	[0.000756 , 0.018025]
Cu	Winter	[0.000468 , 0.002776]	[0.00029 , 0.012077]
	Spring	[0.000589 , 0.004239]	[0.000365 , 0.018412]
	Summer	[0.000983 , 0.003218]	[0.000608 , 0.013976]
	Autumn	[0.000634 , 0.003395]	[0.000392 , 0.014781]
Cr	Winter	[2.03E-05 , 2.58E-05]	[1.19E-05 , 0.000106]
	Spring	[1.97E-05 , 2.76E-05]	[1.15E-05 , 0.000101]
	Summer	[1.65E-05 , 2.43E-05]	[9.6E-06 , 8.85E-05]
	Autumn	[0.000015 , 2.92E-05]	[8.8E-06 , 0.000125]

Table S7. Hazard index (HI) of heavy metals in road dust

Seasons	Adults	Children
Winter	[0.015735 , 0.046324]	[0.009432 , 0.195769]
Spring	[0.013983 , 0.053034]	[0.008389 , 0.21647]
Summer	[0.012377 , 0.026051]	[0.007421 , 0.106727]
Autumn	[0.007698 , 0.026053]	[0.004604 , 0.111163]

Table S8. Carcinogenic risk of heavy metals in road dust via different pathways

Heavy metals	Pathways	Seasons	Adults	Children
Cd	Oral ingestion	Winter	[4.42E-07 , 1.52E-06]	[2.74E-07 , 6.64E-06]
		Spring	[3.87E-07 , 1.03E-06]	[2.39E-07 , 4.47E-06]
		Summer	[8.29E-07 , 1.33E-06]	[5.13E-07 , 5.77E-06]
		Autumn	[6.91E-07 , 1.29E-06]	[4.28E-07 , 5.63E-06]
	Respiratory inhalation	Winter	[1.43E-14 , 4.94E-14]	[2.29E-15 , 5.56E-14]
		Spring	[1.25E-14 , 3.33E-14]	[2.01E-15 , 3.75E-14]
		Summer	[2.69E-14 , 4.30E-14]	[4.30E-15 , 4.84E-14]
		Autumn	[2.24E-14 , 4.19E-14]	[3.58E-15 , 4.72E-14]
	Dermal contact	Winter	[1.52E-09 , 5.26E-09]	[6.99E-10 , 1.70E-08]
		Spring	[1.33E-09 , 2.95E-09]	[6.12E-10 , 1.14E-08]
		Summer	[2.86E-09 , 3.81E-09]	[1.31E-09 , 1.48E-08]
		Autumn	[2.38E-09 , 3.72E-09]	[1.09E-09 , 1.44E-08]
Ni	Respiratory inhalation	Winter	[1.34E-10 , 3.19E-10]	[2.14E-11 , 3.59E-10]
		Spring	[9.20E-11 , 3.98E-10]	[1.47E-11 , 4.48E-10]
		Summer	[9.78E-11 , 2.64E-10]	[1.57E-11 , 2.98E-10]
		Autumn	[8.99E-11 , 2.82E-10]	[1.44E-11 , 3.18E-10]
As	Oral ingestion	Winter	[3.67E-07 , 1.01E-06]	[2.27E-07 , 4.40E-06]
		Spring	[3.33E-07 , 1.18E-06]	[2.06E-07 , 5.15E-06]
		Summer	[2.17E-07 , 4.64E-07]	[1.35E-07 , 2.02E-06]
		Autumn	[1.09E-07 , 4.24E-07]	[6.73E-08 , 1.85E-06]
	Respiratory inhalation	Winter	[8.87E-11 , 2.44E-10]	[1.42E-11 , 2.75E-10]
		Spring	[8.05E-11 , 2.86E-10]	[1.29E-11 , 3.22E-10]
		Summer	[5.26E-11 , 1.12E-10]	[8.41E-12 , 1.26E-10]
		Autumn	[2.63E-11 , 1.02E-10]	[4.21E-12 , 1.15E-10]
	Dermal contact	Winter	[9.26E-08 , 2.55E-07]	[4.25E-08 , 8.23E-07]
		Spring	[8.40E-08 , 2.49E-07]	[3.86E-08 , 9.63E-07]
		Summer	[5.49E-08 , 9.78E-08]	[2.52E-08 , 3.78E-07]
		Autumn	[2.74E-08 , 8.92E-08]	[1.26E-08 , 3.45E-07]
Pb	Oral ingestion	Winter	[2.62E-09 , 3.32E-08]	[1.62E-09 , 1.45E-07]
		Spring	[5.97E-09 , 5.30E-08]	[3.69E-09 , 2.31E-07]
		Summer	[1.81E-09 , 7.11E-09]	[1.12E-09 , 3.10E-08]
		Autumn	[1.54E-10 , 8.54E-09]	[9.53E-11 , 3.72E-08]
	Respiratory inhalation	Winter	[1.42E-12 , 1.80E-11]	[2.27E-13 , 2.03E-11]
		Spring	[3.24E-12 , 2.87E-11]	[5.19E-13 , 3.24E-11]
		Summer	[9.83E-13 , 3.86E-12]	[1.57E-13 , 4.35E-12]
		Autumn	[8.36E-14 , 4.64E-12]	[1.34E-14 , 5.22E-12]

Supporting information:

1. Interval number method

Due to the complexity and fuzziness of environmental health risk assessment, the calculation and evaluation using the mean concentration and single parameter value of experimental analysis into formula may lead to biased conclusions [23]. Based on the literature review, triangular fuzzy number (TFN) and Monte Carlo simulation are commonly used uncertainty control methods. Compared with the Monte Carlo simulation method, the triangular fuzzy number has better applicability in processing poor and low-precision data [24]. At the same time, due to budget control, detailed investigation of exposure parameters may be time-consuming labor-intensive, and difficult to promote [25]. Therefore, this study adopts TFN to conduct quantitative analysis and control of uncertainty and carries out a follow-up discussion on parameter sensitivity. The triangular fuzzy number is defined as A fuzzy number $\tilde{A}^{\alpha}(a_1, a_2, a_3)$ on the real number field R, and a membership function $\mu_{\tilde{A}(x)}$ is defined with the range of [0,1].

$$\mu_{\tilde{A}} = \begin{cases} 0 & x < a_1 \\ \frac{x-a_1}{a_2-a_1} & a_1 \le x < a_2 \\ \frac{a_3-x}{a_3-a_2} & a_2 \le x \le a_3 \\ 0 & x > a_3 \end{cases} \quad (1)$$

where a_1, a_2, and a_3 are non-negative real numbers, which are the minimum, maximum expected value, and maximum value respectively. The α truncated set technique is used to simplify the calculation, and the calculation process is as follows:

$$\tilde{\boldsymbol{A}}^{\boldsymbol{\alpha}} = [a_L^{\alpha}, a_R^{\alpha}] = [(a_2 - a_1)\alpha + a_1, -(a_3 - a_2)\alpha + a_3] \quad (2)$$

where α is the confidence level, which is usually 0.9. $\tilde{\boldsymbol{A}}^{\boldsymbol{\alpha}}$ represents the number of fuzzy intervals of the fuzzy number $\tilde{\boldsymbol{A}}$ under confidence. The four algorithms of fuzzy numbers are as follows [26]:

$$\tilde{A}_1^{\alpha} + \tilde{A}_2^{\alpha} = [a_{L1}^{\alpha} + a_{L2}^{\alpha}, a_{R1}^{\alpha} + a_{R2}^{\alpha}] \quad (3)$$

$$\tilde{A}_1^{\alpha} \div \tilde{A}_2^{\alpha} = [a_{L1}^{\alpha} \div a_{R2}^{\alpha}, a_{R1}^{\alpha} \div a_{L2}^{\alpha}] \quad (4)$$

$$\tilde{A}_1^{\alpha} \times \tilde{A}_2^{\alpha} = [a_{L1}^{\alpha} \times a_{L2}^{\alpha}, a_{R1}^{\alpha} \times a_{R2}^{\alpha}] \quad (5)$$

$$k\tilde{A}_1^{\alpha} = [ka_{L1}^{\alpha}, ka_{R1}^{\alpha}] \quad (6)$$

After the triangular fuzzy number is introduced, the final health risk is also a fuzzy interval, as calculated by the above formula. The acceptable risk threshold for carcinogenic risk is often discussed compared to non-carcinogenic risk with a threshold (HQ<1). It is generally believed that when the carcinogenic risk is less than 1.0E-6, the carcinogenic risk CR can be ignored, but when the CR is greater than 1.0E-4, the carcinogenic risk is more significant in the population [27] [28]. To assist decision-makers in risk management more intuitively, Li et al. (2017) [29] and Xu et al. (2018) [30] established a classification method for carcinogenic risk: I is a very low and negligible carcinogenic risk (-∞, 1.0E-6); II is low carcinogenic risk, [1.0E-

6, 1.0E-5); III is moderate carcinogenic risk, [1.0E-5, 5.0E-5); IV is high carcinogenic risk, [5.0E-5, 1.0E-4); V is very high carcinogenic risk, [1.0E-4, 1). Relative to these risk levels [CRL*, CRR*], the membership function is defined as follows:

$$M = \frac{|[CR_L, CR_R] \cap [CR_L^*, CR_R^*]|}{|[CR_L, CR_R]|} \tag{7}$$

where *M* is the membership degree of the interval [CRL, CRR] to the risk level [CRL*, CRR*].

2. Health risk assessment model

$$INTAKE_{ing} = \frac{C \times ED \times EF \times IngR}{BW \times AT} \times 10^{-6} \tag{1}$$

$$INTAKE_{inh} = \frac{C \times ED \times EF \times IinhR}{BW \times AT \times PEF} \tag{2}$$

$$INTAKE_{dermal} = \frac{C \times ED \times EF \times SA \times SSAR \times ABS}{BW \times AT} \times 10^{-6} \tag{3}$$

where $INTAKE_{ing}$ is oral pathway exposure dose of toxic metals in road dust (mg·/(kg·d)). *C* is the concentration of bioaccessible toxic metals. *ED* is the exposure time (a). *EF* is the frequency of the body's exposure to the medium (d/a). *IngR* is the rate at which people ingest dust into their bodies via the oral pathway (mg/d). *BW* is the weight (kg). *AT* is the exposure time of recipient population (d). $INTAKE_{inh}$ is respiratory pathway exposure dose of toxic metals in road dust. *IinhR* is the average daily air intake (m^3/d). *PEF* is the soil particulate matter release factor (m^3/kg). $INTAKE_{dermal}$ is dermal contact pathway exposure dose of toxic metals in road dust. *SA* is the surface area of exposed skin (cm^2/d). *SSAR* is the surface soil adhesion coefficient (mg/cm^2). *ABS* is the skin absorption factor (dimensionless).
